# The presence of PD-1 positive tumor infiltrating lymphocytes in triple negative breast cancers is associated with a favorable outcome of disease

**DOI:** 10.18632/oncotarget.23717

**Published:** 2017-12-27

**Authors:** Gero Brockhoff, Stephan Seitz, Florian Weber, Florian Zeman, Monika Klinkhammer-Schalke, Olaf Ortmann, Anja Kathrin Wege

**Affiliations:** ^1^Department of Gynecology and Obstetrics, University Medical Center Regensburg, Regensburg, Germany; ^2^Institute of Pathology, University Hospital Regensburg, Regensburg, Germany; ^3^Center for Clinical Studies, University Hospital Regensburg, Regensburg, Germany; ^4^Tumor Center Regensburg, University of Regensburg, Regensburg, Germany

**Keywords:** triple negative breast cancer (TNBC), PD-(L)1

## Abstract

Triple negative breast cancer patients have a poor course of disease not least because of limited treatment options however immunotherapy by targeting the PD-1/PD-L1 checkpoint system is a promising strategy to improve the outcome. Here we systematically investigated the expression of PD-1 on tumor infiltrating lymphocytes and PD-L1 on both tumor and infiltrated immune cells. Moreover, the PD-L1 gene status in tumor cells was assessed.

103 tissue microarray samples derived from triple negative breast cancer specimens were immunohistochemically stained against PD-1 and PD-L1. Dual marker fluorescence *in-situ* hybridization was applied to the PD-L1 gene and centromere region of chromosome 9. The disease free and overall survival rates were determined as a function of the PD-1/PD-L1 status.

A slight gain of the PD-L1 gene region was found in 55% of all samples but an elevated PD-L1/cen9 ratio was rather rare (7%). An increased gene dose is not associated with an enhanced protein expression and the PD-L1 expression only weakly correlates with the amount of immune cell infiltration. Instead, we found an association of PD-L1 expression on tumor and immune cells, respectively. Notably, the PD-1 expression on immune cells is associated with a favorable disease free and overall survival. PD-1 expression indicates an enhanced immunological anti-tumor activity and represents a favorable prognostic impact. A deeper understanding of factors that affect the regulation and function of the PD-1/PD-L1 system is required to establish predictive variables and to utilize the system for therapeutic intervention of triple negative breast cancer patients.

## INTRODUCTION

Triple negative breast cancers (TNBC) account for 10–17% of all breast cancers (BC), tend to grow more aggressively than other subtypes, show relatively early recurrence and intrinsically have poor prognosis [1]. Due to the lack of estrogen receptor (ER), progesterone receptor (PR), and HER2 receptor expression therapeutic options are limited to appropriate cytotoxic treatments. However, due to its enhanced immunogenicity TNBC represent a sub-entity that is apparently predestined for an immunotherapeutic intervention, e.g., an anti-immune checkpoint treatment.

Immunotherapy research is trying to overcome the cancer’s ability to resist the immunological tumor defense and to stimulate or to reactivate mechanisms that result in regaining immunological effectiveness against cancer. To this end different strategies are being developed, amongst them a specific targeting of molecules that are involved in curbing immune cells. Programmed cell death protein 1 (PD-1) expressed on (activated) T-cells and the corresponding programmed death ligand 1 (PD-L1)expressed on immune and tumor cells represent a prominent inhibitory immune checkpoint system that has been demonstrated to play a major role for example in malignant melanoma [2, 3] and squamous non-small cell lung cancer [4, 5]. An immune checkpoint treatment has already been FDA approved for these entities. In contrast, the immune checkpoint targeting in BC patients is being evaluated but not yet part of the approved therapeutic portfolio [6].

Due to its higher genetic instability, an enhanced mutational load, and the appearance of neoantigens PD-L1 expression is more frequently found in HER2-positive and triple negative BCs than in other BC sub-entities (e.g., the luminal cohorts) [7]. Moreover, PD-L1 expression has been associated with the degree of tumor infiltrating lymphocytes (TILs) [8–10]. However, systematic analyses addressing the PD-1/PD-L1 system in BC are rare. First data from the KEYNOTE-012 (NCT01848834) study revealed a clinical activity of an anti-PD-1 IgG4, namely pembrolizumab [10]. More specifically, in 27 PD-L1-positive (pre-treated) TNBC patients, the application of pembrolizumab achieved an overall response rate of 18.5%, although only patients with PD-L1-pos. tumors (cut off: ≥ 1% PD-L1-pos cells) were included. Other clinical trials especially addressing TNBC and HER2-pos. patients are ongoing [6, 11, 12]. The GeparNuevo trial (NCT02685059), for instance, evaluates the therapeutic efficacy of the PD-L1 antibody MED14736 (AstraZeneca) in combination with a taxane/anthracycline based cytotoxic treatment of TNBC (https://clinicaltrials.gov/ct2/show/NCT02685059). Interestingly, even patients with PD-L1-negative scored tumors seem to benefit from an anti-PD-L1 treatment. Even though it is known that PD-L1 expression can be triggered as response to a T-cell attack, the underlying molecular / cellular mechanisms contributing to the treatment response require elucidation. It appears plausible, however, that an efficient inhibition of an immunological tumor defense by PD-L1-positive tumor cells requires interaction with PD-1-positive lymphocytes. Thus, a systematic assessment of both parts of the PD-1/PD-L1 system on tumor cells and TILs will shed light on the tumor tissue related immune status and might reveal a valuable prognostic or predictive impact.

Here we scored the amount of TILs in 103 TNBC samples and immunohistochemically evaluated the expression of PD-L1 on tumor cells and lymphocytes. In addition we analyzed the PD-1 expression on TILs and quantified the PD-L1 gene copy number in tumor cell nuclei by fluorescence-*in-situ*-hybridization (FISH). We associated and correlated these parameters to each other and retrospectively analyzed the overall and progression free survival (OS, PFS) of TNBC patients as a function of PD-1 and PD-L1. Overall, we found a favorable outcome of TNBC patients with PD-1 positive TILs compared to those patients who had tumors with lymphocytes expressing low levels or no PD-1.

## RESULTS

### An increased PD-L1 gene copy number or PD-L1/cen9 hybridization ratio is rare

**Figure 1:**
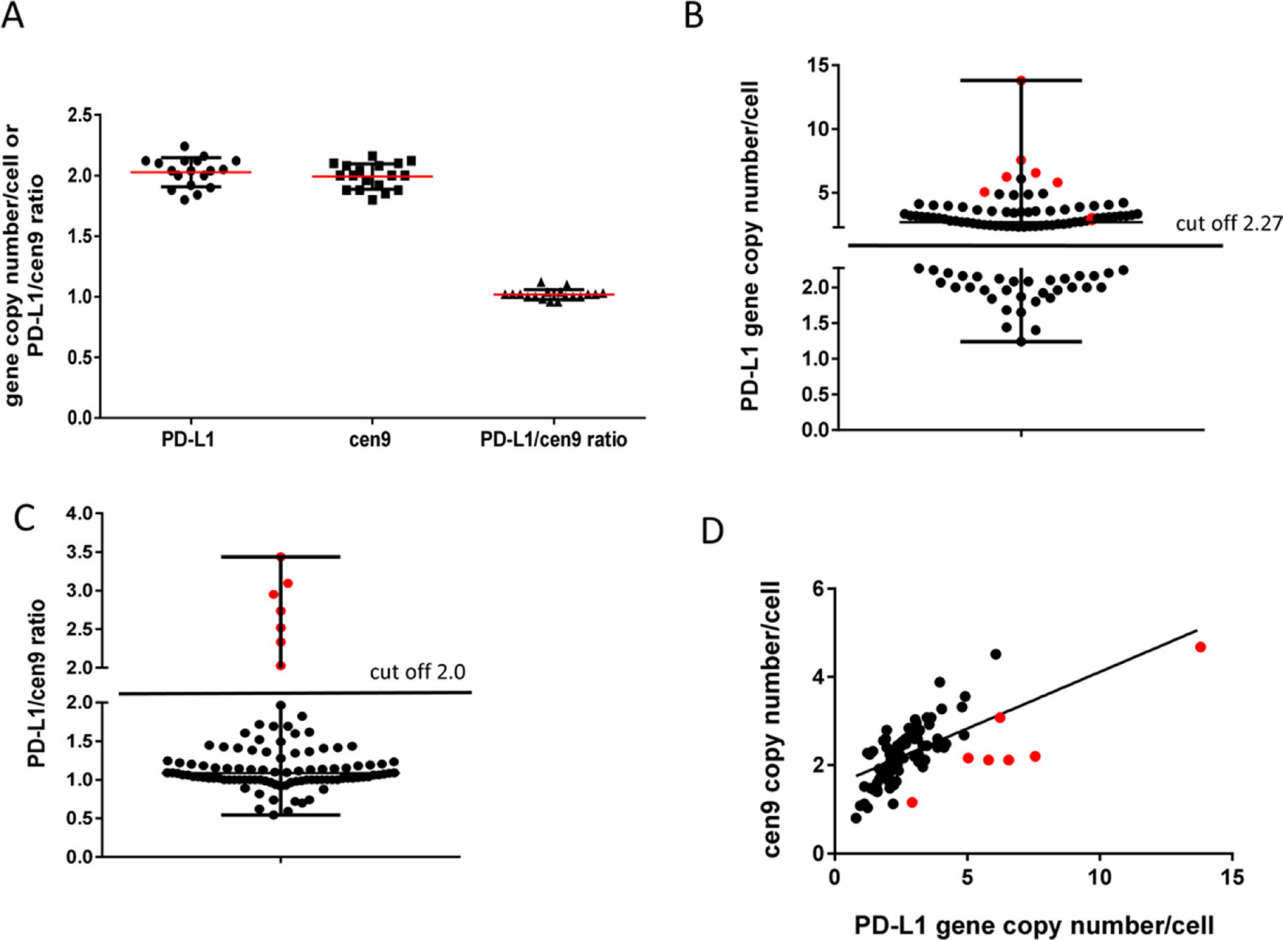
PD-L1 gene amplification (FISH) in TNBC patients (**A**) determination of PD-L1, centromere copy number and ratio in benign breast tissue (mean +/– SD*; n* = 18). Threshold for abnormal gene amplification/loss were estimated by the calculation: mean +/– 2× SD. (**B**) 103 TNBC were analyzed using the cut off 2.27 PD-L1 gene/1 cell determined in (A) and grouped in PD-L1 increased (*n* = 59 (57%)) and PD-L1 normal/decreased (*n* = 44 (43%)) samples. (**C**) TNBC patients were separated into PD-L1 gene amplified (> 2 (ratio); *n* = 7 (7%)) and not altered (< 2 (ratio); *n* = 96 (93%)). (**D**) Correlation between PD-L1 gene and centromere copy number per cell were determined using Pearson’s correlation coefficient (*r* = 0.652; *p* < 0.001; *n* = 103). Red symbols in (B, C and D) refer to 7 samples with PD-L1/cen9 ratio >2.0.

To evaluate the range of variation in normal tissue and to estimate the threshold for pathological gene amplification, we analyzed PD-L1 and cen9 gen copy numbers in 18 benign mammary tissues derived from breast cancer reduction surgeries (Figure 1A). In those tissues we found the PD-L1 gene copy number within the range of 1.79–2.27 (SD = 0.12, mean = 2.03). The mean of cen9 hybridization was 1.99 (SD = 0.11) and ranged between 1.77 and 2.21. Accordingly, the mean of the PD-L1/cen9 ratio in healthy tissue was 1.02 (SD = 0.04) and was within the range of 0.94–1.10. These thresholds were applied to define copy number alterations in breast cancer tissues. We found 59/103 (57%) samples with increased (> 2.27) PD-L1 gene copy numbers whereas 44/103 (43%) were below the amplification threshold (Figure 1B). Off note, 5 patients were even below the average mean of healthy donors and are considered to carry a PD-L1 gene loss (copy number < 1.8). Most often, a slightly increased PD-L1 gene copy number occurs simultaneously with a likewise moderately increased cen9 copy number which results in a PD-L1/cen9 ratio within the normal range (Figure 1C). In only seven samples we revealed a significantly enhanced PD-L1/cen9 ratio (≥ 2.03) which is supposed to represent a moderate but real gene amplification (Figure 1B–1D). A strong association between the PD-L1 and cen9 gene copy number was validated by the calculated Pearson’s correlation coefficient (*r =* 0.652; *p* < 0.001; Figure 1D). A PD-L1 gene copy gain, a loss, and a simultaneously increased PD-L1 and cen9 copy number are exemplarily shown in Supplementary Figure 1A, I–III, respectively. No correlation between the PD-L1 gene copy number and the OS (*p* = 0.87) or PFS (*p* = 0.62) could be revealed (Supplementary Figure 1B and 1C).

### An increased PD-L1 gene copy number in tumor cells is not associated with an enhanced PD-L1 protein expression

**Figure 2:**
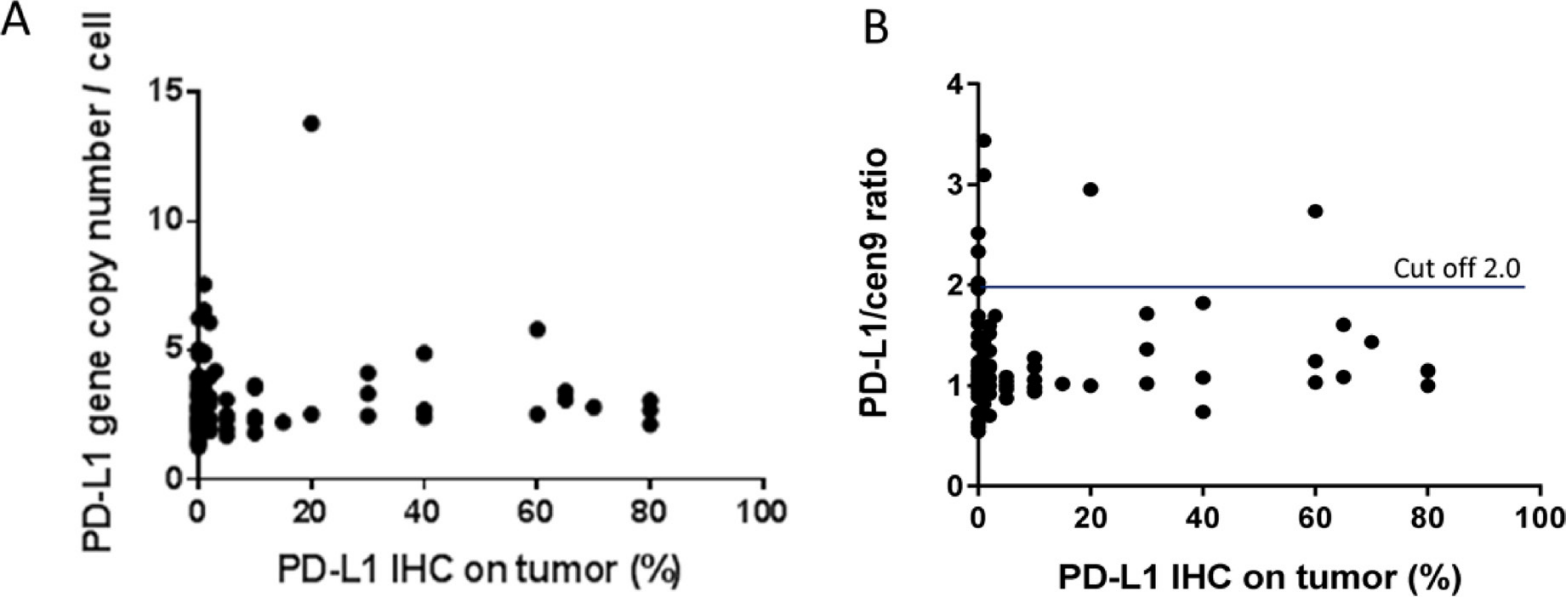
PD-L1 gene amplification and PD-L1 protein expression in TNBC (**A**) There is no correlation between PD-L1 gene copy number (*r* = 0.053; *p* = 0.607; *n* = 97) (**B**) nor a correlation between PD-L1/cen9 ratio (*r* = 0.087; *p* = 0.397; *n* = 97) and the PD-L1 expression (%) on tumor cells. Correlation was measured using the Pearson correlation coefficient.

PD-L1 positive tumor cells were found in 55/97 (57%) of all specimens. However, in 37/55 of all positive samples the frequency of positive cells was below 10%. Notably, no correlation was found between the PD-L1 gene copy number and PD-L1 expression (*r* = 0.053; *p* = 0.607; *n* = 97; Figure 2A) and not between the PD-L1/cen9 ratio and PD-L1 positive tumor cells (*r* = 0.087; *p* = 0.397; *n* = 97; Figure 2B). PD-L1 positive TILs were found in 71/98 (72%) of all cases. Five samples of immunochemically stained and PD-L1 gene/cen9-hybridized tissue specimens are exemplarily shown in Supplementary Figure 2.

### PD-L1 expression on tumor cells is associated with PDL-1 expression on immune cells but without significant impact on OS or PFS

**Figure 3:**
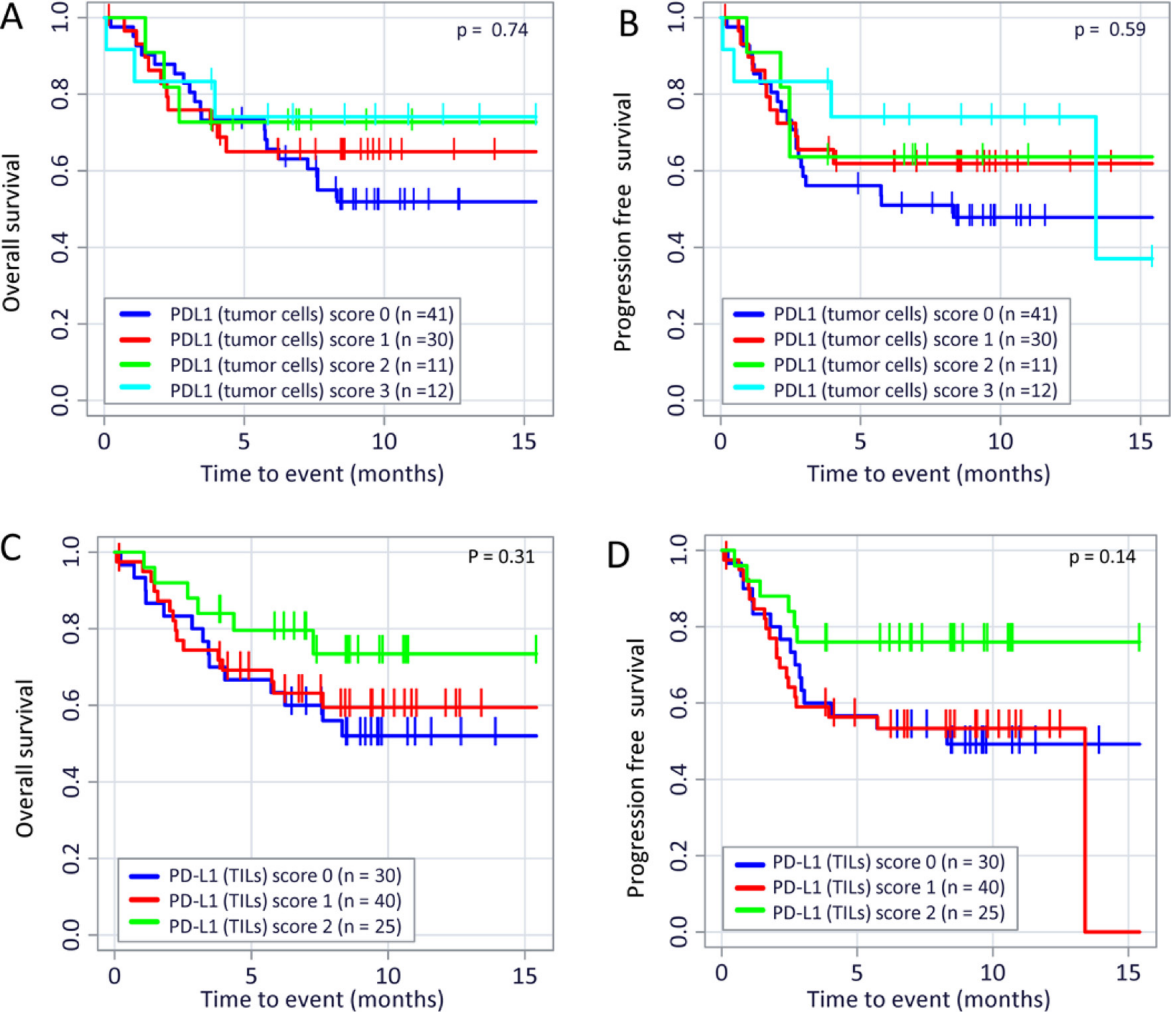
PD-L1 expression on TILs or tumor cells and its correlation to OS and PFS in TNBC patients Kaplan–Meier overall survival (OS; (**A**) *p* = 0.87) and progression free survival (PFS; (**B**) *p* = 0.62) curves in patients with different PD-L1 expression on tumor cells are displayed. There were also no correlation detectable between overall survival (OS; (**C**) *p* = 0.31) and progression free survival (PFS; (**D**) *p* = 0.14) in patients with different PD-L1 expression on TIL. The *p* values were calculated using the log-rank test (Mantel-Cox).

We calculated a (weak) correlation between PD-L1 expression on tumor cells and on TILs (*p* < 0.01; Spearman-Rho facto*r* = 0.455; Table 1). Nevertheless, PD-L1 expression on tumor cells does not significantly affect the OS (Figure 3A; *p* = 0.74) or the PFS (Figure 3B; *p* = 0.59) of the patients. Complementary, the PD-L1 expression on TILs does also not correlate with OS (*p* = 0.31) or PFS (*p* = 0.14) as shown in Figure 3C and 3D. In addition, the TIL score does not correlate with the PD-L1 expression on tumor cells (Spearman-Rho = 0.227; data not shown). Moreover, the TIL score (Supplementary Figure 3) is not significantly associated with an increased OS (*p* = 0.17) or PFS (*p* = 0.13). Only nine samples with low infiltration (Score 1 = 1–9 TIL/HPF) but 43 tissues scored 2 (10–49 TIL/HPF) and 50 scored 3 (>50 TIL/HPF) were identified. Overall, 93/102 (91%) of all samples showed high (Score 2) or a very high (Score 3) immune cell infiltration. Therefore, only a trend towards a better outcome of disease for Score 3 cases (compared to Score 1/2 cases) could be revealed (Supplementary Figure 3).

**Table 1:** PD-L1 expression on tumor cells and immune cells in TNBC

			PD-L1 IHC score (tumor cells)				
			0	1	2	3	total
**PD-L1 IHC Score (TILs)**	**0**	*n* (%)	25 (24.3%)	7 (6.8%)	1 (1.0%)	0 (0.0%)	33 (32.0%)
	**1**	*n* (%)	16 (15.5%)	16 (15.5%)	4 (3.9%)	7 (6.8%)	43 (41.7%)
	**2**	*n* (%)	6 (5.8%)	9 (8.7%)	7 (6.8%)	5 (4.9%)	27 (26.2%)
	**total**	*n* (%)	47 (45.6%)	32 (31.1%)	12 (11.7%)	12 (11.7%)	103 (100.0%)

The correlation between PD-L1 expression on TIL and PD-L1 expression on tumor cells are displayed as number and % in groups belonging to both scores. The overall correlation between both characteristics were measured using the Spearman correlation coefficient (Spearman-Rho = 0.455; *p* < 0.01).

### The presence of PD-1 positive TILs correlates with the presence of PD-L1 tumor cells and is associated with an improved OS and PFS

**Figure 4:**
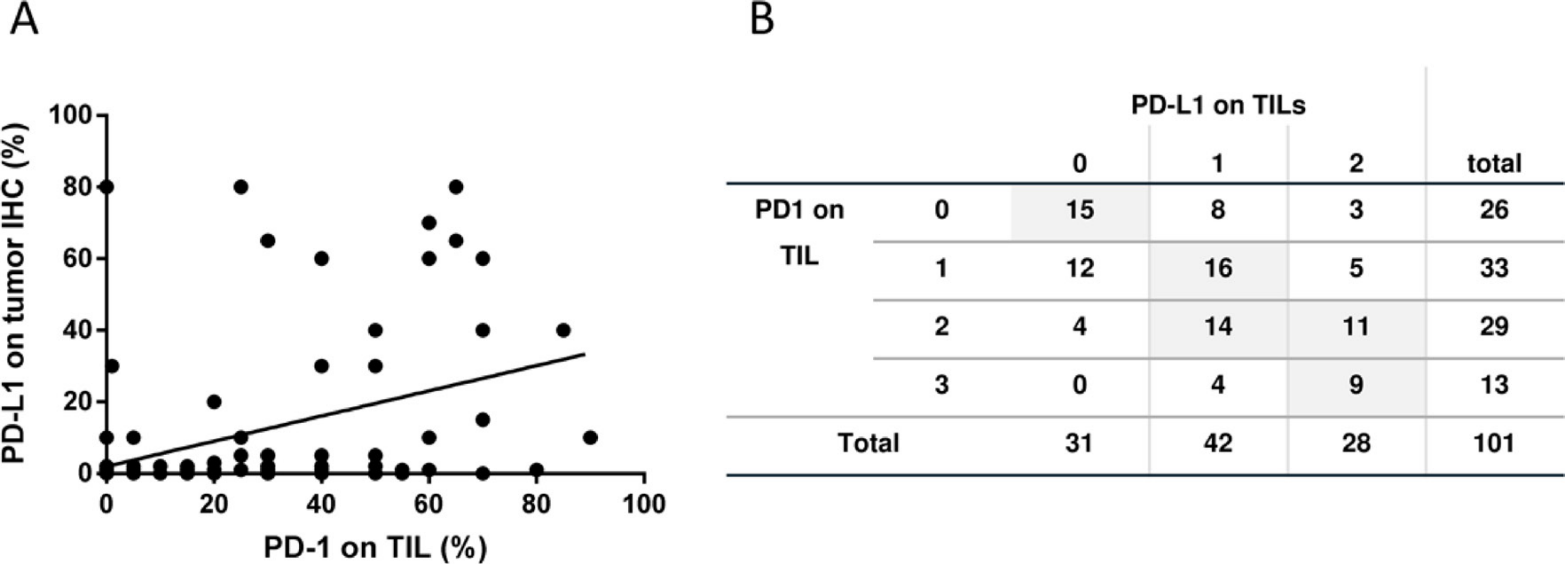
PD-1 and PD-L1 expression on tumor and immune cells (**A**) Correlation between PD-L1 expression on tumor cells and PD-1 expression on TIL is displayed (person rho = 0.469; *p* < 0.001; *n* = 99). (**B**) The correlation between PD-L1 and PD-1 expression on TIL are displayed as number and % in groups belonging to both scores (Spearman-Rho = Spearma *n* = 0.493 (*p* < 0.001, *n* = 101).

**Figure 5:**
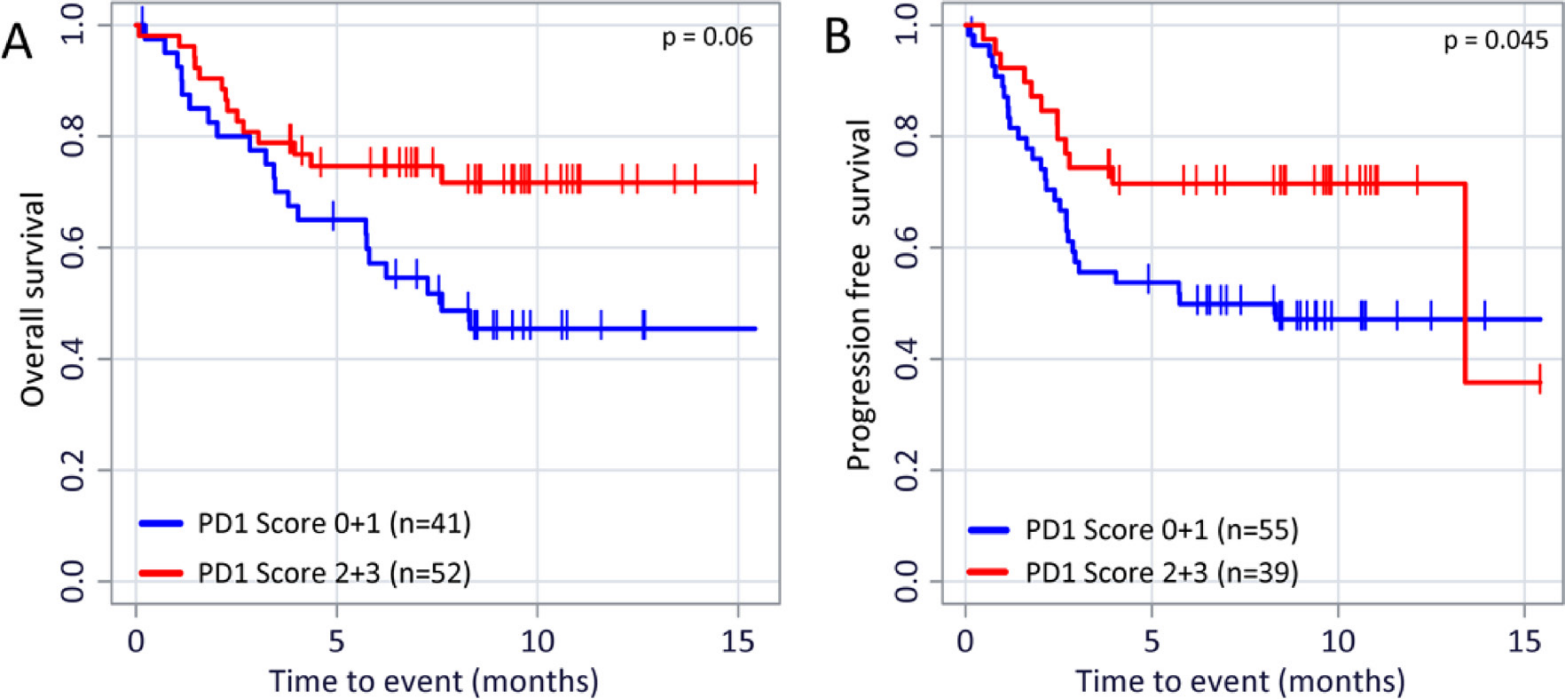
PD-1 expression on TILs and its relevance on OS and PFS Kaplan–Meier overall survival (**A**) *p* = 0.06) and progression free survival (**B**) *p* = 0.045) curves in patients with different PD-1 expression are displayed. The *p* values were calculated using the log-rank test (Mantel-Cox).

Despite the fact that the TIL score could not be correlated to PD-L1 expression nor to the patient’s OS and PFS the presence of PD-1 positive TILs favorably affects the outcome of disease: On the on hand we found a direct correlation of PD-1 expression on TILs and PD-L1 expression on tumor cells (Figure 4A; *r* = 0.469 (*p* < 0.001), *n* = 99) and between PD-1 and PD-L1 expression on TILs (Figure 4B; *r* = 0.493 (*p* < 0.001); *n* = 101). On the other hand, and even more importantly, the PD-1 expression on TILs has a favorable impact on the OS (Figure 5A; *p* = 0.06) and especially on the PFS (Figure 5B; *p* = 0.045). The PD-1 expression turned out as the strongest prognostic marker that determines the outcome of disease.

## DISCUSSION

Aim of this study was to evaluate the PD-1/PD-L1 status on tumor and immune cells in TNBC specimens both on the genomic and protein level. Data sets were correlated mutually and to the outcome of disease.

We assessed the PD-L1 gene and cen9 copy numbers in tumor cells of 103 TNBC tissues by dual marker FISH and interrelated the cytogenetic data to the respective PD-L1 expression. We found 59/103 (57%) events with a rather moderate increase of PD-L1 gene copy numbers. Not more than 7/103 (7%) specimens showed an elevated PD-L1 gene copy number only (i.e., no increased cen9 numbers) which results in a (slightly) enhanced PD-L1/cen9 ratio. In contrast, 96/103 samples (93%), including those with moderately enhanced PD-L1 gene copy numbers, come without an increase of the PD-L1/cen9 ratio which suggests the absence of PD-L1 gene amplification in these cases. Since the PD-L1 gene region is located very much distal on the short arm of chromosome 9 (i.e., 9p24.1) and far away from the cen9 region a common amplification of both regions can be excluded in samples that show both moderately enhanced PD-L1 and cen9 copy numbers. Instead, the correlation of simultaneously elevated PD-L1 gene and cen9 copy numbers (*r* = 0.65), which does not result in an increased PD-L1/cen9 ratio, indicates the presence of (low grade) polysomy 9. Moreover, the missing correlation between PD-L1 gene copy numbers and PD-L1 expression on tumor cells suggests that the PD-L1 gene dose does not determine the degree of protein expression. In other words an enhanced PD-L1 protein expression was found independently from the PD-L1 gene copy number and the PD-L1/cen9 ratio. Unlike as, for example, the expression of the HER2 receptor protein, which (in BC) is strongly determined by the her2 gene copy number/ gene amplification, the PD-L1 expression seems not to be chromosomally determined. This is consistent with other studies in which only a weak correlation of PD-L1 transcripts and no correlation of PD-L1 protein content with PD-L1 gene copy number aberrations were reported [13, 14]. Moreover, the PD-L1 expression seems to be not directly triggered by the presence of lymphoid immune cells. Instead, there is evidence suggesting that PD-L1 expression is rather regulated by a variety of alternative mechanisms, amongst them the activity of signaling pathways, transcriptional factors, epigenetic factors, and a number of microRNAs [15]. PD-L1 has been described to be multifactorial and in particular dynamically regulated. For example, PD-L1 negative tumor cells might permit T-cells to invade into the tumor tissue. However, PD-L1 expression can be induced by IFNγ released by these activated T- or NK cells. Vice versa, a PD-L1 expression by tumor cells can impede (further) T-cell infiltration and INFγ release that might entail reduced PD-L1 expression. Thus, the PD-L1 phenotype is most likely not stable but affected by multiple factors and is rather independent from the inherent PD-L1 gene copy number.

Within the cohort of TNBC patients subjected to this study PD-L1 does not significantly affect the course and outcome of disease neither when expressed on tumor cells nor on immune cells. Even though an association between PD-L1 expression and longer survival has been described elsewhere for non-gynecological malignancies e.g., metastatic melanoma [16] as well as merkel cell [17] and colorectal carcinomas [18]. The PD-L1 related survival data of BC patients are greatly inconsistent [19]. A number of studies performed on basal-like or TNBC reported a positive correlation between PD-L1 expression and a favorable prognosis [13, 20, 21]. However, a reverse correlation between PD-L1 expression and prognosis has been also described [22–24]. Overall, it appears evident that the regulation of PD-L1 expression does not underlie a simple unidimensional factor but is regulated in a rather complex and multifactorial manner. A number of potential factors contributing to the regulation of PD-L1 expression on tumor cells are discussed in more detail elsewhere [16]. For example, the presence of PD-L1 positive (tumor) cells is considered as an indicator of immunosuppression (caused by an immune cell attack) but might also imply a tumor response to endogenous inflammatory activity [16, 15]. As indicated by a preclinical study [26] and a very recent analysis on 58 patients with early (and mainly hormone receptor positive) BC patients in a neoadjuvant setting [27] the PD-L1 expression on tumor and stromal cells can change (either increase or decrease) upon cytotoxic treatments. However, the same study reported that - similar to our findings - neither the initial PD-L1 expression nor the modified expression in residual compared to primary tumors had an effect on patient’s outcome [27].

We found that the PD-L1 expressions on tumor and immune cells do correlate, which is in good agreement with other reports on non-small cell lung cancer [28] and even BC [29]. The finding suggest that the immunosuppressive environment is determined by both immune and tumor cells. More importantly, we revealed a correlation of tumor cell related PD-L1 and immune cell related PD-1 expression, which indicates a tumor-immune-cell interaction and an antigen-induced and TILs mediated anti-tumor immune pressure. An induction of the PD-1/PD-L1 checkpoint system and associated pathways by activated CD8^+^ and INFγ has been previously observed in a melanoma based murine model [30]. Although incompletely effective, recruitment of TILs to the tumor site (e. g., by chemotactic attractants) might have induced a partial antitumor activity that can explain our observation. More specifically, PD-1 expression on antigen-experienced CD8^+^ T-cells, which had contact to PD-L1-pos. tumor or immune cells, might represent a T-cell phenotype characterized by impaired effector function and a persistent expression of inhibitory receptors, a phenomenon, which has been termed “T-cell exhaustion” [31]. The presence of formerly or currently activated PD-1 positive immune cells is supposed to reflect some degree of immunological tumor defense which in turn might favorably affect the course of disease. Indeed, we revealed a prolonged OS as a function of PD-1 positivity. More precisely, the higher the TIL associated PD-1 score the better the OS. This finding substantiates the interpretation that an antigen-induced antitumor immune pressure raises a recruitment of immune cells to the tumor site that results in a partially successful antitumor defense.

The presence of TILs in particular in HER2-positive and TNBC samples has been repeatedly associated with a favorable prognosis [32, 33] and, not less importantly, with an improved response to neoadjuvant cytotoxic [34, 35] and target specific [24, 36, 37] tumor treatments. However, the infiltrated immune cells have been rarely sub-classified or phenotyped. Bottai *et al.* found PD-1 (and LAG-3) positive TILs in 15% of TNBCs and an association of PD-1 expression with the presence of CD8^+^ cytotoxic T-cells [33]. Here we report that not only the presence of TILs in general but in particular PD-1 positive TILs (conceivably T-cells) have a significant favorable impact on the outcome of TNBC disease. A number of studies undertaken on BC and other tumor entities (e.g., head and neck cancer) concord with this finding [38], whereas others do not [39–41]. Considering, that the immunohistochemical assessment of PD-1 on TILs is inherently a “snap shot” at a given time, PD-1 positivity might either represent the active state of lymphocytes (when analyzed relatively early during the carcinogenesis and progression) or reflect an already expired lymphocyte activity (exhaustion upon interaction with PD-L1). Taking a temporal regulation into account might explain discrepancies within reports. Overall, the prognostic impact of PD-1 expression on TILs (in BC and other malignancies) remains uncertain and subject of complex temporal and multifactorial regulation. Extended analyses are required that include additional parameters involved in this regulation.

We could not reveal a correlation of an increasing amount of TILs (expressed by the TIL score ranging from 0 to 3) with the PFS or OS. This is probably due to the fact that most of the tumor samples came with a rather enhanced immune cell infiltration (i.e., 93/103 (90%) samples had an infiltration score of 2 or 3). Tissue specimens without the presence of TILs (Score 0) were not observed at all. In this study we did not differentiate lymphocyte subsets. However, PD-1 expression can basically be found on T-, NK-, and B-cells, but also on monocytes and even regulatory T-cells (T_regs_). It has been reported that PD-1-pos. T_regs_ represent impaired activity [42]. A reduced activity of T_regs_ can cause an increased activity of effector T-cells and consequently stimulate the systemic immune response. A tumor cell associated PD-L1 and T_reg_ associated PD-1 interaction, which would impair a T_reg_ mediated inhibition of e. g., cytotoxic T-cells, could explain the favorable impact of PD-1 which entails an improved outcome of disease [25]. Although, our data did not reveal a direct correlation between the tumor cell associated PD-L1 expression and disease outcome a correlation between the PD-1 and PD-L1 expression became obvious which might support the afore outlined interpretation.

A drawback of our study might be the use of TMAs (instead of total tissue specimens) since immune cell infiltration can be heterogeneous and vary amongst different tissue areas. Consequently, the size of specimens that undergoes the investigation potentially plays a role for proper evaluation of immune cell infiltration and the estimation of PD-1 and PD-L1 expression [25]. One might expect, for example, that PD-L1 positive tumor cells can predominantly be found close to the TILs rather than in non-infiltrated areas. However, no relationship has yet been demonstrated between the tissue size and PD-L1 expression nor between time since sample collection and IHC staining [43] and the use of TMAs for immunochemical PD-1/PD-L1 analyses is not uncommon [41]. Here the selection of a tissue area for inclusion into this study was performed under supervision of a pathologist who screened the available tissue totally and thereby made sure not to oversee potential immune cell infiltration. Based on this procedure we found 90% of all samples to be TIL positive, though to a different extent.

Overall, the prognostic value of both PD-L1 and PD-1 expression in TNBC (and probably other taxonomic BC entities) remains uncertain and requires further investigation. Notwithstanding, data supporting either a favorable or an adverse effect of the PD-1/PD-L1 system on the course of disease should not necessarily be contradictory since different effects of PD-1/PD-L1 might be elicited by the environment and the type of immune cell that express this receptor and its ligand. Hence, it is rather unlikely that the assessment of PD-L1 only (expressed on tumor cells) will decisively facilitate a patient stratification in respect of eligibility for a checkpoint treatment. Instead, the data heterogeneity amongst a great number of studies suggests that multifactorial analyses are required to understand the impact of PD-1/PD-L1 positive cells with tumor tissues on immunological defense, tumor growth and progression and finally the course and outcome of disease. Further studies that comprise not only the overall-evaluation of TILs and the degree of PD-L1 expression on tumor and immune cells but also include the differential analysis of PD-1 expression on immune cell subpopulations (i.e., NK-, dendritic, CD4- and CD8-positive T-cells, and monocytes) will specify and thus considerably enhance the diagnostic and prognostic significance of immune cell analyses [24]. Moreover, additional biomarkers such as the TIL formation, the presence of neoantigens presented by HLA molecules or soluble factors in the microenvironment could be informative. Only differential / multiplex analyses of the regulation of PD-1 and PD-L1 expression on tumor and immune cells will assure any prognostic and predictive impact.

## MATERIALS AND METHODS

### TNBC patient database

103 TNBC tissue samples were derived upon surgery and were recruited between the years 2004 to 2015. 90.5% of those samples, which were derived from cytotoxically treated patients, were taken at the non-pretreated stage (Table 2). The triple negative status was (immuno-)histochemically determined based on the estrogen/progesterone receptor, Ki67, and Her2-receptor status and the grading, and if applicable by FISH, determined by pathological diagnostics at the University of Regensburg. Clinico-pathological parameters were documented by the institute of pathology and the breast cancer center of the university cancer center Regensburg (Table 2). Clinical follow up was correlated with the data from the Tumor Centre Regensburg a population-based regional cancer registry covering a population of more than 2.2 million people including Upper Palatinate and Lower Bavaria. The documentation comprises individual patient data, information on primary diagnosis, treatment regimens, course of disease, and the complete follow-up. Benign control tissues were taken from healthy women who underwent breast reduction.

**Table 2:** Basic demographic data of 103 evaluable TNBC cases (BCT = breast conserving therapy)

Clinico-pathological parameter	(*n*)	(%)
**Tumor stage**		
I	24	23.3
II	52	50.4
III	12	11.7
IV	7	6.8
unknown	8	7.8
**Histologic subtype**		
invasive ductal	90	87.3
invasive lobular	0	0
medullary	12	11.7
mucinous	1	1
**Grading**		
1	1	1
2	19	18.4
3	77	74.8
unknown	6	5.8
**Mean age at diagnosis: 53.4y**		
premenopausal	43	41.7
postmenopausal	54	52.4
unknown	6	5.8
**Surgery**		
mastectomie	41	39.8
BCT	62	60.2
**Radiation**		
yes	70	68
no	29	28.2
unknown	4	3.8
**Chemotherapy**		
a: yes	84	81.6
b: adjuvant	76	90.5 (of a)
c: neoadjuvant	8	9.5 (of b)
d: no	11	10.7
e: unknown	8	7.8

### Fluorescence *in-situ* hybridization and imaging

FISH was performed as described recently [13]. In brief 3–4 µm thick deparaffinized TMA specimens were pretreated in 98°C 0.01N Na-Citrate buffer for 30 min, incubated with pepsin (ZytoVision Ltd., Bremerhaven, Germany) for 5 min at 37°C, and washed with Millipore water followed by ethanol dehydration (70, 80, and 100%). Subsequently, ten µl of the original probe were added on each specimen and slides were covered by a cover glass and fixogum rubber cement. After a denaturation step (5 minutes at 73°C), slides were incubated over night at 37°C. Finally, the cover glass was removed, the samples were washed, and 4′,6-diamidino-2-phenylindole (DAPI) nuclear counter staining was added according to the manufacturer’s instruction.

Fluorescence *in situ* hybridization was performed using the directly labeled PDCD1LG2/cen9 dual color probe (ZytoVision GmbH, Bremerhaven, Germany). The PDCD1LG2 specific probes were labeled with SpectrumGreen and the cen9 specific probe with SpectrumOrange. PDCD1LG2 hybridization spots reflect the PD-L1 gene copy number whereas the cen 9 spots are considered to reflect the number of chromosome 9 within a cell nucleus.

Sealed slides were imaged with an AxioImager Z1 fluorescence microscope (Zeiss, Oberkochen, Germany) equipped with specific filter sets for DAPI fluorescence (excitation 360 ± 20 nm, emission 460–25 nm), SpectrumGreen (excitation 480 ± 15 nm, emission 535 ± 20 nm), SpectrumOrange (excitation 538 ± 10 nm, emission 575 ± 15 nm). Hybridization signals in 25 non-overlapping cell nuclei per specimen were quantified by two independent observers, and count values were averaged. If necessary, brightfield microscopy was used to verify the presence of either malign or benign breast tissue in the visual field. Analyzes were performed using AxioImager-Z1 (Zeiss) and the hybridization signals of 50 non-overlapped nuclei were manually counted on single cell basis. Results are presented as PD-L1 gene signals per one cell and calculated as FISH ratio (PD-L1 gene signals/chromosome 9 signals).

### Tissue embedding and manufacture of tissue microarrays

All specimens were acquired from the tissue archive of the Institute of Pathology, University of Regensburg (Germany). The embedding procedure was performed as described elsewhere [44]. Immediately after surgery, the breast tissues were transferred into the formalin fixative (4% formaldehyde, 1% sodium phosphate; SG Planung, Holzkirchen, Germany). The total fixation time was between 12 h (min.) and 36 h (max.). The specimens were then subjected to automated dehydration and paraffin immersion. Tissue dehydration was performed by subjecting the tissues to a series of ascending ethanol concentrations (70% for 30 min, 70% for 60 min, 96% for 60 min, 96% for 50 min, 100% for 50 min, and 100% for 90 min), and was completed by incubation in 100% xylene (2 × 50 min). Finally, the tissues were embedded in paraffin by the use of a Shandon Hypercenter XP (2 × 30 min; 2 × 60 min).

Tissue microarrays (TMAs) were generated as described previously [45] and were used for PD-L1 immunohistochemistry and PD-L1/cen9 FISH analyses. In brief, for each tumor a representative tumor section was selected from a hematoxylin and eosin (H&E)-stained section of the donor block. The relevant tissue section was identified by a pathologist based on tissue architecture, morphology and HER2-IHC. Core cylinders with a diameter of 1.5 mm each were punched from this area and deposited into a recipient paraffin block; 4 µm TMA sections were mounted on charged slides (SuperFrost Plus; Menzel, Braunschweig, Germany) and used for FISH analysis. H&E-stained TMA sections were used for reference histology.

### PD-L1 immunohistochemistry and bright field microscopy

1.5 µm paraffin sections were prepared from the embedded tissue blocks. Specimens were deparaffinized and pretreated by microwave heating for 30 min at 320 W in 0.1 M citrate buffer adjusted to pH 7.3. The immunostaining was automatically performed on a Ventana Nexes autostainer (Ventana, Tucson, USA) by using the streptavidin– biotin peroxidase complex method and 3,3′-diaminobenzidine (DAB) as chromogen. The autostainer was programmed based on the instructions given by the iView DAB detection kit (Ventana). The mouse monoclonal anti-PD-1 antibody NAT105 (ab52578) and the rabbit monoclonal anti-PD-L1 28–8 (ab205921) were used (both abcam, Cambrindge, MA, USA). The specimens were microscopically analyzed using a Zeiss Axiovert 200 instrument (Zeiss). The degree of immmune cell infiltration and the frequency of positive immune/tumor cells were scored by percentage or by number of cells/high power fields (HPF) and translated into a score system ranging that covers the scores 0, 1, 2, and 3 (Tables 3–6).

**Table 3:** PD-L1 score applied to tumor cells

Score	% of PD-L1+ tumor cells
0	0
1	1–9
2	10–39
3	>40

**Table 4:** TIL score applied to tumor tissues (HPF = high power field)

Score	# TIL/HPF
0	absent
1	<10
2	10–50
3	>50

**Table 5:** PD-L1 score applied to TILs (HPF = high power field)

Score	# of PD-L1+ TIL /HPF
0	absent
1	<10
2	10–50
3	>50

**Table 6:** PD-1 score applied to TILSon TIL

Score	# of PD-1+ TIL /HPF
0	absent
1	<10
2	10–50
3	>50

### Statistical analyses

Survival curves were estimated using the Kaplan–Meier method, and the log-rank test was used to test for differences between the groups. To calculate the association between parameters, Pearson or Spearman correlation coefficient analyses were applied. All reported *P*-values were two-sided. *P* values less than 0.05 were considered significant. All statistical analyses were performed using R version 3.3.3 (The R Foundation for Statistical Computing) or GraphPad Prism (Ver. 6, GraphPad Software, La Jolla, CA, USA).

## SUPPLEMENTARY MATERIALS FIGURES


