# Supplementary material for: The presence of PD-1 positive tumor infiltrating lymphocytes in triple negative breast cancers is associated with a favorable outcome of disease
Source: Oncotarget. 2017 Dec 27;9(5):6201–12. doi: 10.18632/oncotarget.23717 (PMC5814205; doi:10.18632/oncotarget.23717)
Supplement: Supplementary file 1 [file oncotarget-09-6201-s001.pdf]

The presence of PD-1 positive tumor infiltrating lymphocytes in triple negative breast cancers is associated with a favorable outcome of disease

SUPPLEMENTARY MATERIALS

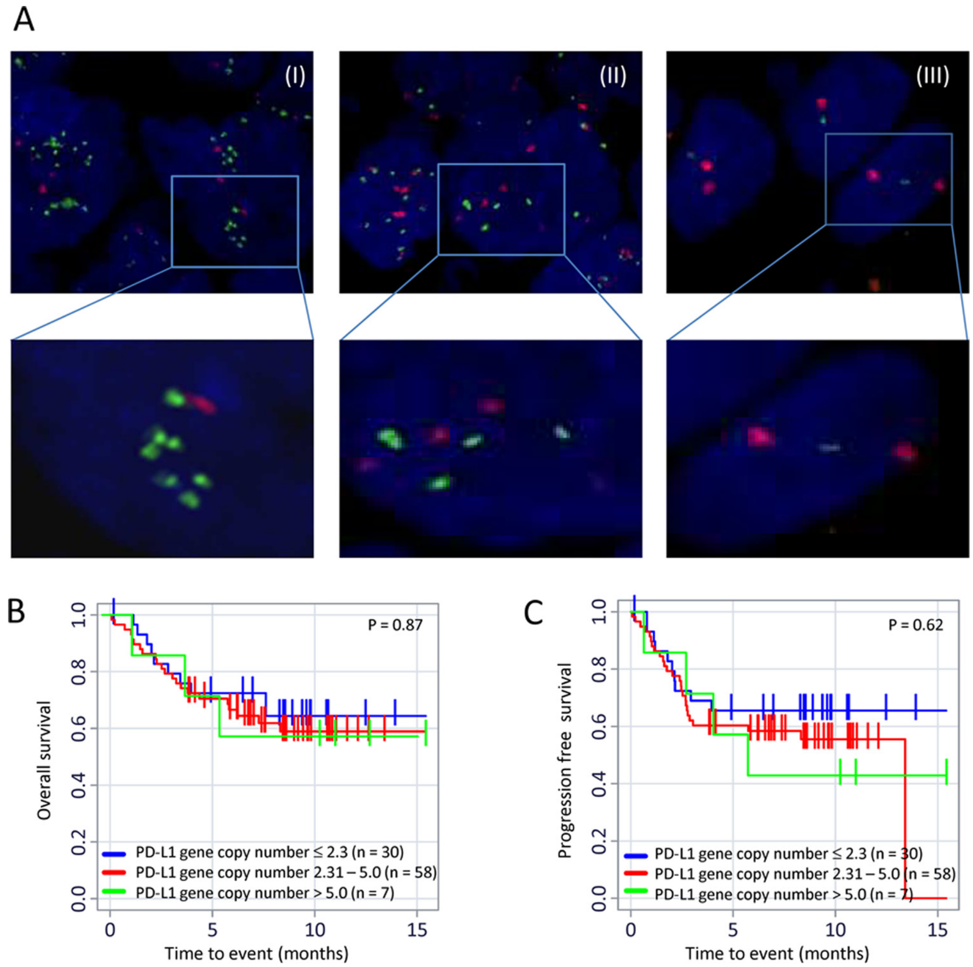

Supplementary Figure 1: PD-L1/cen9 alteration in TNBC patients and the associated outcome. (**A**) Exemplarily cases of PD-L1 (green) and Cen9 (red) gene copy gain, gene loss, and increased ratio are shown. (**B**) Kaplan–Meier overall survival (OS) and (**C**) progression free survival (PFS) curves in patients with PD-L1 gene copy number alteration. The differences were not significant (log-rank test (Mantel-Cox); OS $p = 0.87$, PFS $p = 0.62$).

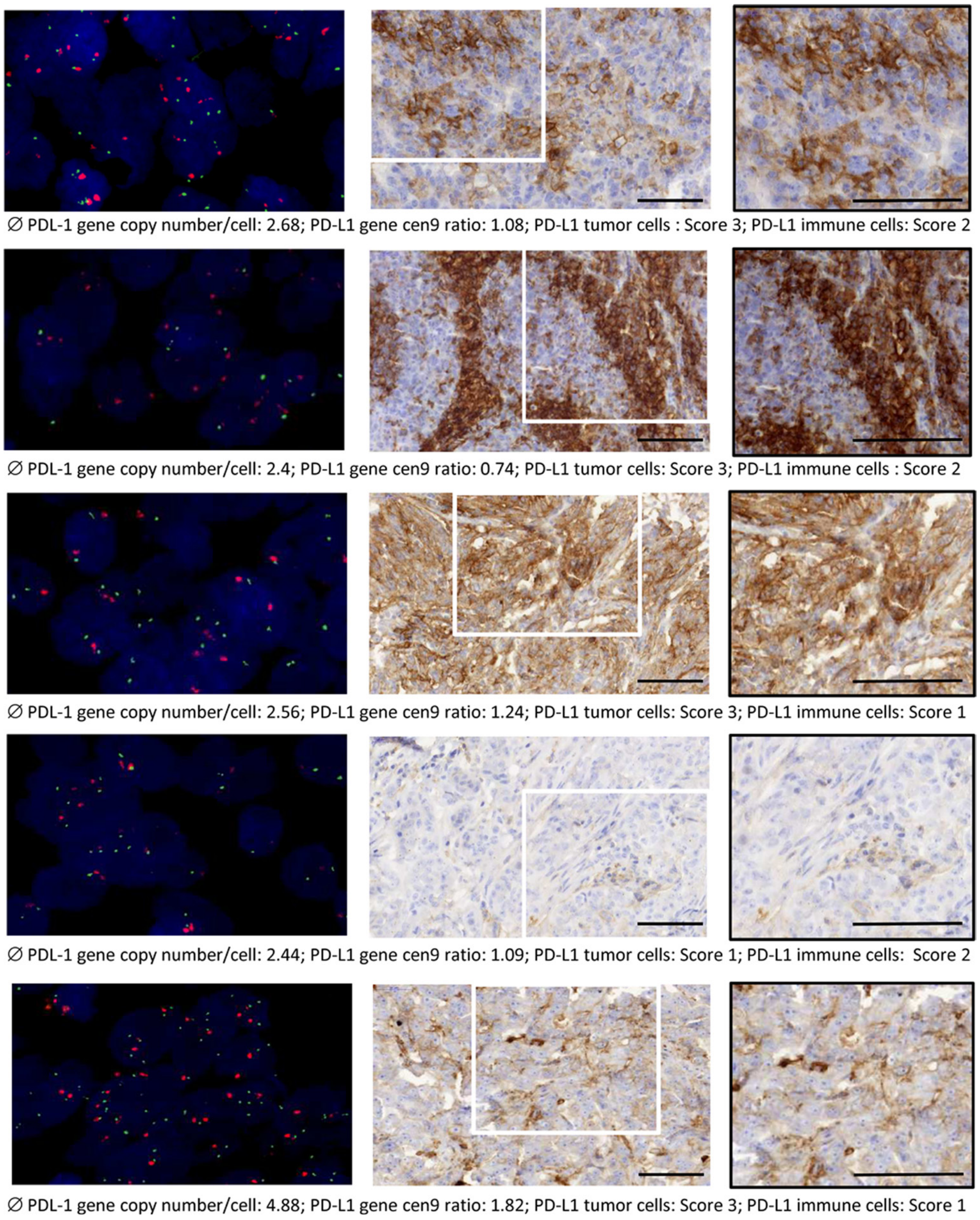

Supplementary Figure 2: PD-L1/cen9 *in-situ* hybridization and PD-L1 expression in TNBC histological sections. The left column displays examples of PD-L1 (green) and cen9 (red) hybridization spots derived from *in-situ* hybridization of a corresponding specimen. Gene and cen9 copy numbers are indicated as well as the gene/cen9 ratio. In the middle column TNBC specimens immunohistochemically stained anti-PD-L1 and categorized by the pathologist are exemplarily shown. Subareas are enlarged and shown in the right column. Implemented bars represent 100 µm.

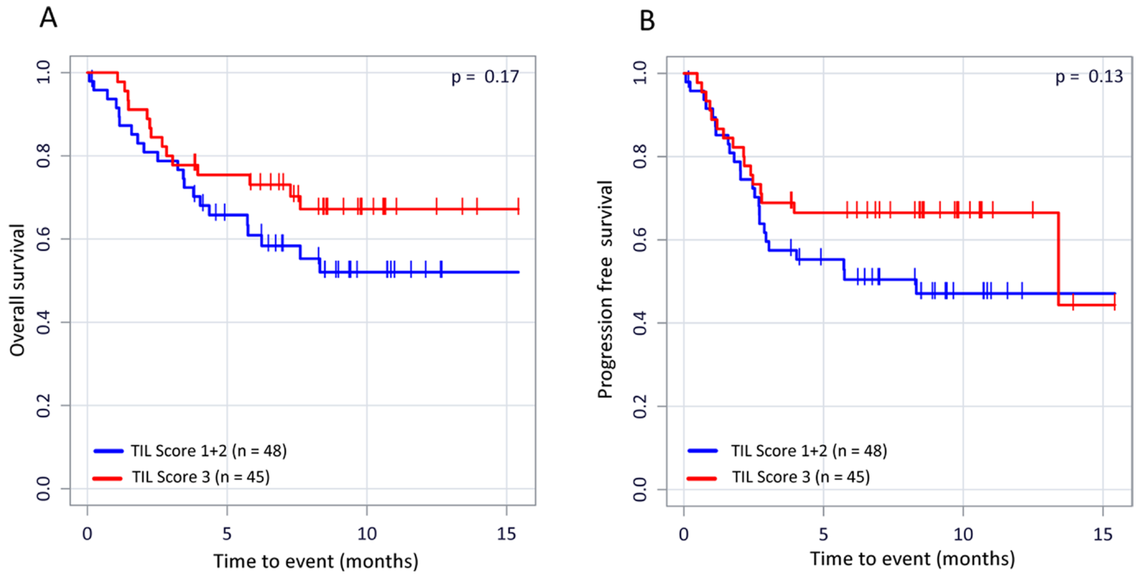

Supplementary Figure 3: TIL score and the corresponding outcome (OS and PFS) in the analyzed TNBC cohort. Kaplan–Meier OS (**A**) and PFS (**B**) curves in patients with different PD-L1 expression on tumor cells are displayed. The *p* values were calculated using the log-rank test (Mantel-Cox).
